# Impact of polyvinyl chloride nano-plastics on the biochemical status of *Oreochromis niloticus* under a predicted global warming scenario

**DOI:** 10.1038/s41598-025-87558-8

**Published:** 2025-01-29

**Authors:** Ahmed Mohamed Soliman, Ayman S. Mohamed, Amr A. Abdel-Khalek, Shereen R. Badran

**Affiliations:** 1 Department of Zoology, Faculty of Science, Cairo University, Giza, Egypt; 2 Department of Physiology, Faculty of Medicine, Tobruk University, Tobruk, Libya

**Keywords:** Global warming, Polyvinyl chloride nano-plastics pollution, *O. niloticus*, Biochemical parameters, Oxidative stress, Freshwater ecology, Environmental sciences

## Abstract

Plastic pollution and global warming are widespread issues that lead to several impacts on aquatic organisms. Despite harmful studies on both subjects, there are few studies on how temperature increases plastics’ adverse effects on aquatic animals, mainly freshwater species. So, this study aims to clarify the potential impact of temperature increases on the toxicological properties of polyvinyl chloride nano-plastics (PVC-NPs) in Nile tilapia (*Oreochromis niloticus*) by measuring biochemical and oxidative biomarkers. The fish groups were subjected to three distinct temperatures (30, 32, and 34 °C) and subsequently separated into two groups: 0 and 10 mg/L of PVC-NPs, as it is expected that these temperatures may modify their chemical properties, which can influence their absorption and toxicity in fish. After 4 days, the biochemical response of fish exposed to PVC-NPs and elevated temperatures showed a significant increase in the levels of plasma total proteins, albumin, globulin, aspartate aminotransferase (AST), alanine aminotransferase (ALT), alkaline phosphatase (ALP), creatinine, and uric acid. Additionally, the level of oxidative stress biomarkers in the liver, gills, and brain was found to have a significant increase in malondialdehyde (MDA) concentration and a decrease in glutathione reduced (GSH) concentration and catalase (CAT) activity in all studied groups. Finally, the current findings revealed a synergistic cytotoxic effect of PVC-NPs and temperatures on the metabolic and oxidative stress indices of *O. niloticus*.

## Introduction

Global warming has increased dramatically over the last few decades. Changes in the world’s heat regimes are known to influence the stability of ecosystems, human society, and the economy^1^. Recent studies predict that by 2100, the average world surface temperature will rise by 1.5 to 5.8 °C, causing intense heat waves that will have a global negative impact on freshwater, terrestrial, and marine ecosystems^2^. Variations in water temperature have significant effects on fish’s physiological functions, including early development, growth, metabolism, reproduction, and behavior, and even small increases in the current temperatures of aquatic environments will impact many fish species^3^. In addition to interfering with an organism’s physiological processes, raised temperatures can alter the distribution and biological consequences of chemical toxicity^4^. Besides, plastic pollution poses a significant threat to many ecosystems worldwide, particularly freshwater habitats^5^. In several studies, plastic debris has been found in water, sediment, zooplankton, fish, bivalves, and crustaceans^6–9^. When plastic pollution is combined with the issue of global warming, it can modify their chemical properties, which can impact their environmental fate and influence their absorption, accumulation, and toxicity in aquatic species^2,10–13^. According to Shams et al.^14^, global plastic production is estimated to be 400 million tons per year, with yearly growth rates exceeding 4%. plastics are used in many aspects of daily life, such as industries, packaging, vehicles, electronics, clothing, and agricultural items^15^. The large plastic waste that enters aquatic environments in various ways can degrade into tiny fragments known as micro or nano-plastics, which have emerged as a significant environmental issue in recent years^16^. This degradation may occur naturally owing to weathering from sun, wind, and waves; erosion from water movement; or from human activities^17^. Nano-plastics (NPs) are synthetic polymeric matrix elements that are small, typically smaller than 100 nm, solid and water-insoluble particles that cause a hazard effect on aquatic life^18^. Discharge from industrial effluent, wastewater treatment plants, surface runoff, and transportation through rivers can release NPs into aquatic environments as primary sources, or they can become secondary sources after plastic degradation by physical, chemical, and biological processes^19,20^. Because of the small size of NPs, aquatic animals can get them easily into their circulatory systems through eating, respiration, or skin penetration. After that, they are dispersed; some are probably eliminated, while others reach inside organs and tissues, where they may be retained or accumulate, leading to negative effects on animal health^21,22^. According to Banaee et al.^17^, many studies showed that plastics can impair fish’s behavior, inhibit immune function, change gut microbiota, and cause reproductive issues, inflammation, oxidative stress, genetic toxicity, and tissue damage. Different kinds of plastic polymers have been discovered in various environments^23^. According to Iheanacho et al.^24^, polyethylene (PE), polyvinylchloride (PVC), polypropylene (PP) polyurethane, polystyrene (PS), and polyethylene terephthalate (PET) are most prevalent in the global environments. PVC is one of the most valuable products of the chemical industry and represents the largest worldwide market for plastics, even though an assessment of the environmental and health hazards of plastic polymers based on polymer chemical composition has placed PVC at the top of the list of hazardous polymers in the environment^25^. They are frequently utilized in pipeline systems for the water and sewage sectors’ plastic pressure pipe systems and are also widely used in building materials, construction, and home furnishings^26^. PVC has been identified as one of the main newly discovered contaminants of the aquatic environment in recent years^27,28^. Freshwater is essential to the life cycle of plastics; however, compared to marine organisms, less research has been done on the harmful impacts of plastics on freshwater species, especially fish^29^. Considering this, *O. niloticus* was used as an animal model as it is a native species in almost all kinds of freshwater environments; beyond being simple to breed, tolerant to a variety of conditions, and growing quickly, that thrives in warm water, and high market demand^30^. There is a paucity of studies on how temperature increases affect the adverse effects of plastics on aquatic animals, especially freshwater species, despite the existence of toxicity studies on both subjects independently^31^. Furthermore, not much research has been done to determine NPs’ effects compared to MPs on aquatic organisms, particularly higher trophic level species like fish^32^. Therefore, the current study’s specific objectives were to investigate the possible effect of temperature increases on the toxicological properties of PVC-NPs in the most common freshwater fish, *O. niloticus* by measuring biochemical and oxidative biomarkers. Additionally, our results could provide insight into the effects of increasing temperatures and PVC-NPs on freshwater environments and their biota under the predicted global warming scenario.

## Materials and methods

### Characterization of PVC-NPs

PVC-NPs (commercial virgin pre-production in white powders) were obtained from a local PVC compounds producer in Egypt (Misr Gulf Co. for Modern Industries). According to Wang et al.^33^, PVC-NPs powder was subjected to several techniques for various characterizations. Fourier transform infrared spectroscopy (FTIR-4100typeA) was used to confirm the polymer composition of the NPs used. FTIR spectra were investigated at a resolution of 4 cm^-1^ in the 4000 –500 cm^-1^ range. Then, the size and morphology of PVC-NPs were evaluated by transmission electron microscopy (FETEM, JEM-2100 F, JEOL Inc., Japan) at an accelerating voltage of 200 kV. Furthermore, the zeta potential (Nano-Zeta Sizer-HT, Malvern Instruments, UK) analysis was utilized as well to evaluate the PVC-NPs stability in water at experimental room temperature (30 ^◦^C). Additionally, to investigate the interaction between PVC-NPs and increasing temperature, zeta potential was evaluated at 32 °C and 34 °C.

### Fish acclimatization and experimental design

From an uncontaminated fish farm near Kafr El-Sheikh (in Summar, 2023), the 80 mature male fish under investigation were transferred to Cairo University’s Faculty of Science’s ecology lab in well-aerated tanks. After that, for 10 days of acclimation, they were placed in dechlorinated glass aquaria (40 × 70 × 26 cm) with constant aeration in the laboratory conditions. The characteristics of the water were as follows: 28–30 °C (normal lab temperature), pH 7.2–7.4, and 6.6–7.8 mg/L of dissolved oxygen. The water was partially changed (20%) every day to get rid of organic wastes and surplus food. During this period, fish were fed on commercial pellet meals with a dose of 3% of their total weight each day. These meals included crude protein (32%), fiber (5%), fat (4%), ash (13%), and moisture (10%). According to the NRC^34^, fish feed continuously until they appear to be satisfied. Once the fish adaptation period ended, 60 healthy fish with a body length of 13.42 ± 0.94 cm and a weight of 33.86 ± 4.81 g were randomly assigned to five fish per 40 L well-aerated glass aquarium with dimensions of 40 × 70 × 26 cm (12 glass aquaria). Then, fish were exposed to two sets of three temperature regimes (30 °C, 32 °C, and 34 °C) using a thermostat (REI-SEA, 300 watts, Japan) and confirmed by temperature sensor. The selected temperatures related to the environmental temperature in the summer and predicted rising temperatures resulted from global warming^2,10^. The temperatures of water aquaria were achieved with a consistent temperature rise of Δ1°C every 12-h period to not give fish thermal shock or sudden stress^10^. After that, one set of aquaria received 10 mg/L of PVC-NPs, while another set received none. The sub-lethal concentration was selected in agreement with Hamed et al.^35^. So, the experimental groups were 0 mg/L PVC-NPs groups and 10 mg/L PVC-NPs groups at 30 °C, 32 °C, and 34 °C with duplicated aquaria per group for 4 days.

### Fish sampling, plasma collection, and tissue homogenate preparation

Fish were collected randomly after the experimental time and anesthetized by immersion with 20 mg/L clove oil, and overdose (200 mg/L) was used to euthanize the fish^36^. According to Clark et al.^37^, a 1 ml heparinized syringe was used to collect blood samples from a fish’s caudal vein. Then, the blood was collected in clean tubes, centrifuged for 20 min at 4 °C around 5000 rpm to extract plasma, and kept at -80 °C for further biochemical analysis. Additionally, the liver, gills, and brains were removed, rinsed with a cold solution of 0.9% NaCl, and homogenized with (50 mM, pH 7.4) of cold phosphate buffer. Then, they were put in cooled centrifugation at 3000 rpm for 15 min. Afterward, the collected supernatants were preserved at -80 °C, even determining the biomarkers for oxidative stress. The protocol for obtaining plasma and tissue homogenization was based on the manufacturer kit guidelines (Biodiagnostic, Dokki, Giza, Egypt).

### Biochemical analysis

Plasma samples were analyzed using enzymatic colorimetric methods from Biodiagnostic commercial kits (Giza, Egypt). According to Gornal et al.^38^, total plasma protein (g/dL) was measured by the biuret method at 550 nm. This approach is based on the idea that protein forms a compound with copper (Cu^+ 2^) in an alkaline environment. The plasma albumin concentration (g/dL) was determined using the approach outlined by Doumas et al.^39^, which relied on the production of a blue-green albumin/bromocresol complex and its measurement at 630 nm. The plasma globulin concentration was calculated by subtracting the plasma albumin from the total plasma protein^40^.

For indications of liver functions, plasma aspartate aminotransferase (AST) and plasma alanine aminotransferase (ALT) levels (U/L) were measured in accordance with Reitman and Frankel^41^ methodology by conversion of oxalate (α-ketoglutarate) to pyruvate and combination of pyruvate with 2,4-dintrophenylhydrazone (DNPH) and reading at 505 nm. The activity of plasma alkaline phosphatase (ALP) (U/L) was also determined by measuring the conversion of phenyl phosphate to phenol and phosphate at 510 nm. This measurement was performed in the presence of 4-aminophenazone and potassium ferricyanide, following the method reported by Belfield and Goldberg^42^. Additionally, plasma creatinine and uric acid concentrations (mg/dL) were determined using the procedures provided by Bartels et al.^43^ and Barham and Trinder^44^, respectively, for assessing kidney functions. The plasma creatinine method was dependent on its reaction with picric acid in an alkaline solution, giving a colored complex that was read at 495 nm. Moreover, uric acid measurement was based on uricase, which transforms uric acid into allantoin and hydrogen peroxide. Then hydrogen peroxide, in the presence of the peroxidase enzyme, oxidizes 4-aminoantipyrine and 3,5-dichloro-2-hydroxybenzinesulphonate to create a colored quinoneimine solution reading at 510 nm.

### Determination of oxidative stress biomarkers

The obtained supernatant from tissue homogenates (liver, gills, and brain) was used for evaluating the concentration of MDA^45^, GSH^46^, and the activity of the CAT enzyme^47^. All oxidative stress indicators were measured according to the instructions of commercial kits (Biodiagnostic, Dokki, Giza, Egypt). In brief, the MDA concentration in the samples (nmol/g. tissue) was quantified by measuring the strength of the MDA-TBA pink color product at 534 nm after the reaction of the samples in the acidic medium at 95 °C with thiobarbituric acid (TBA). Furthermore, the method used to quantify GSH involved reducing 5,5′-dithiobis (2-nitrobenzoic acid) in samples with glutathione. This produced a yellow molecule whose absorbance at 405 nm is proportional to the GSH content (mmol/g. tissue). For CAT determination, samples were dispersed with phosphate buffer, followed by hydrogen peroxide (H_2_O_2_), and the reaction was inhibited by a catalase inhibitor. Then, the colored chromophore was produced after adding 4-aminophenazone and 3,5-dichloro-2-hydroxybenzene sulfonic acid to the solution in the presence of peroxidase, whose levels were inversely proportional to CAT quantity in the samples (U/g. tissue) which determined at 510 nm.

### Statistical data analysis

The data was initially arranged in an Excel spreadsheet and then examined using the IBM SPSS 20 statistical program (Chicago, IL) at the 0.05 significance level. A student t-test was employed to analyze variations between non-exposed and exposed PVC-NPs groups with regard to each temperature. At the same time, one-way analysis of variance (ANOVA) was used to analyze the variation of the studied temperature groups for each that received PVC-NPs or not. Duncan’s multiple-range test was employed to determine the similarities between various groups. Additionally, a two-way ANOVA was used to assess the significance between PVC-NPs toxicity, exposure to different temperatures, and interaction effects. All data were expressed as mean ± SE and.

## Ethics statement

All methods were performed in accordance with the relevant guidelines and regulations. The present investigation was conducted in compliance with ARRIVE guidelines and approved by the Institutional Animal Care and Use Committee (IACUC), Faculty of Science, Cairo University, with approval no. (CUIF 3023)

## Results

### Characterization of PVC-NPs

**Fig. 1:**
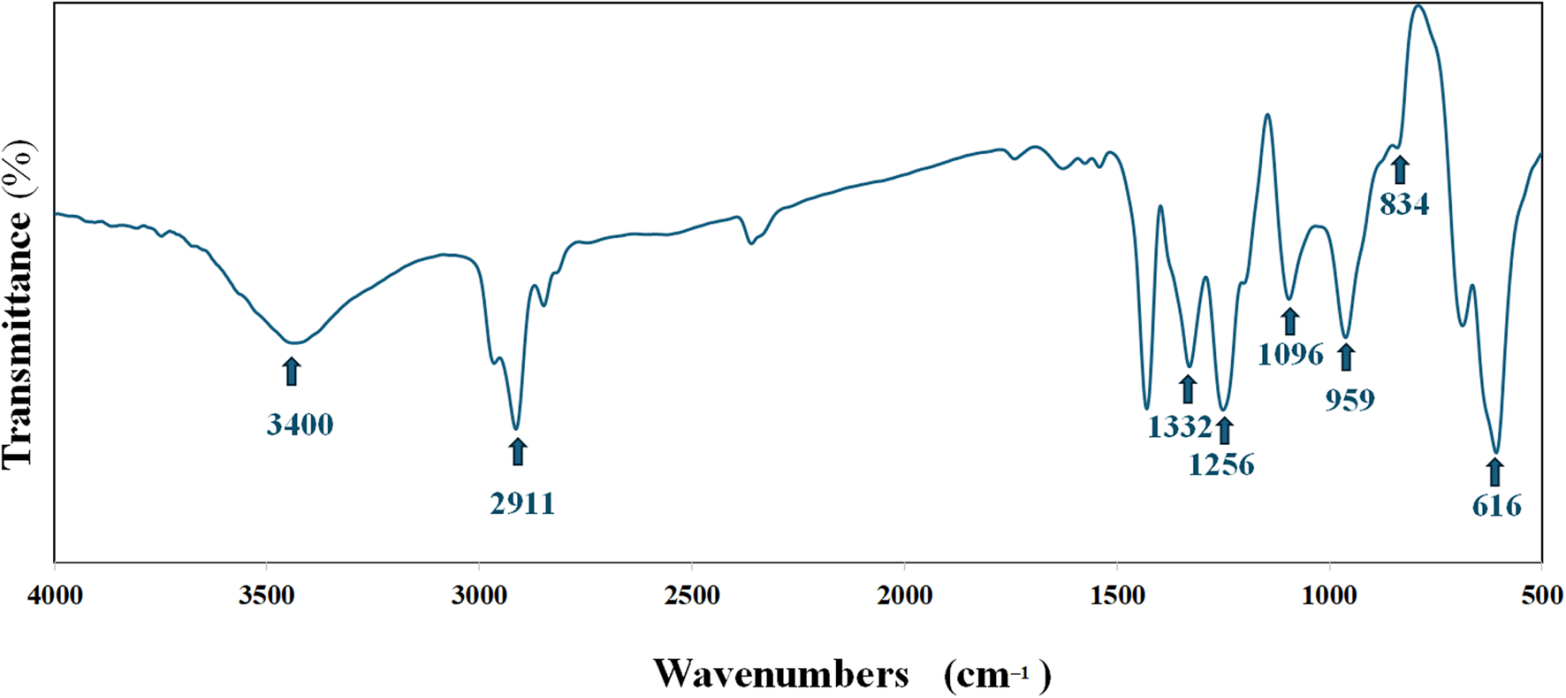
Fourier-transform infrared spectroscopy (FTIR) spectrum of PVC-NPs.

**Fig. 2:**
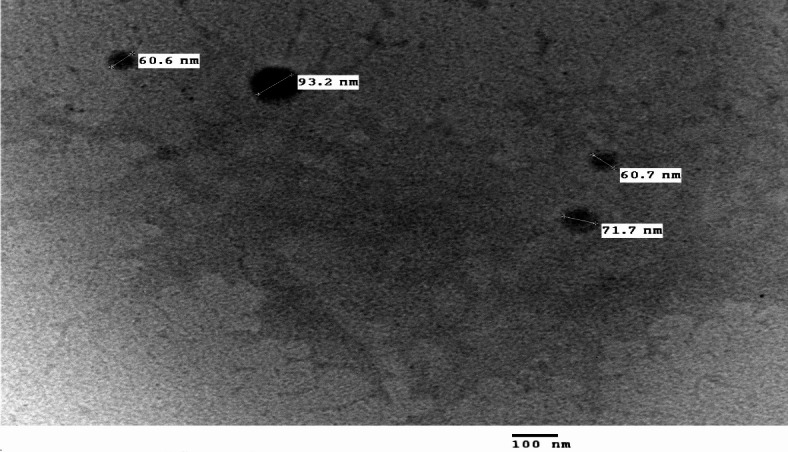
Transmission electron microscopy (TEM) image of PVC-NPs.

**Fig. 3:**
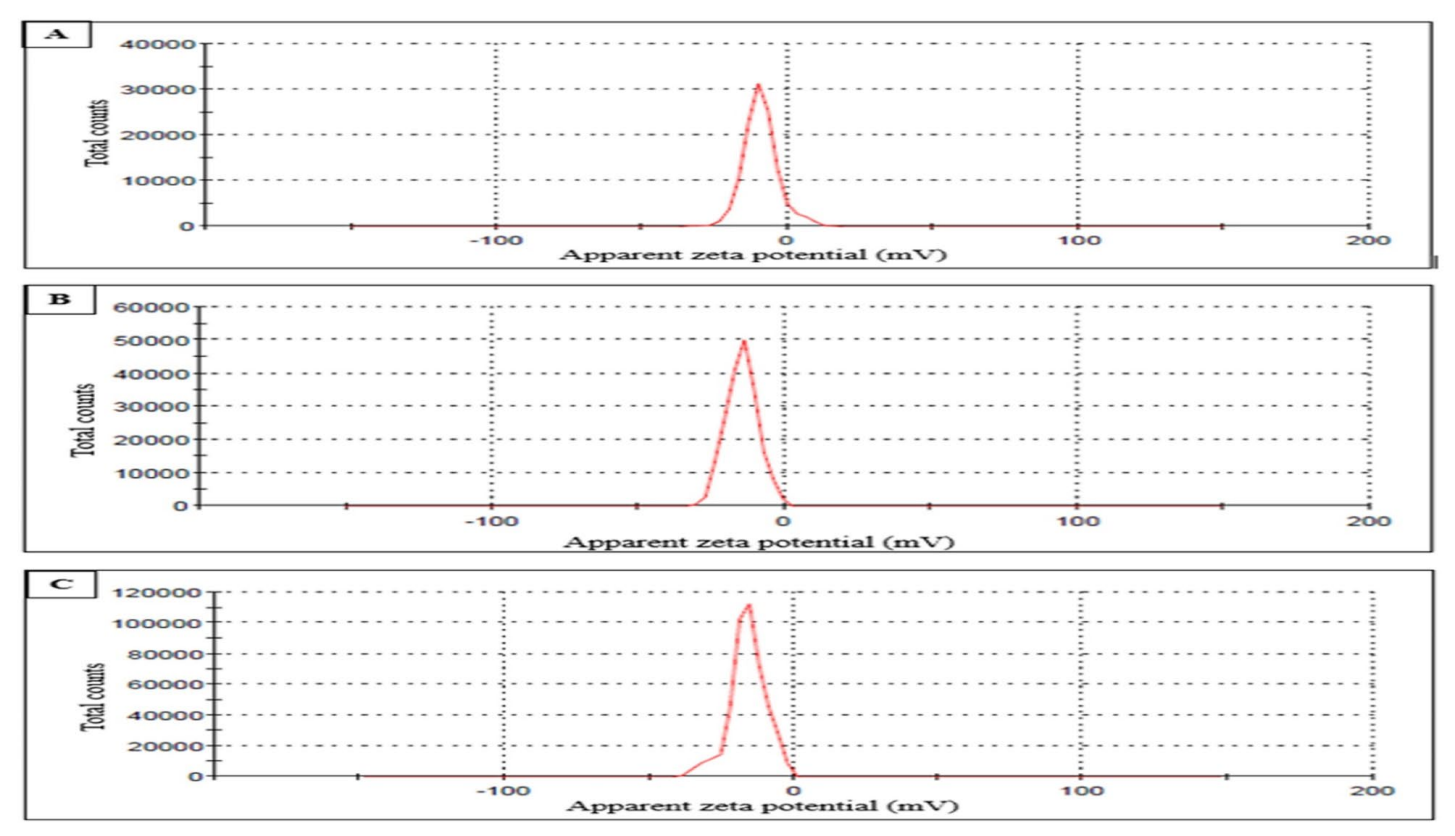
The zeta potential distributions of PVC-NPs at water temperatures (**a**) 30 °C; (**b**) 32 °C, and (**c**) 34 °C.

The FTIR spectrum for PVC-NPs is shown in Fig. 1, which assisted in the detection of chemical bonding in NPs. The vibrational bands observed at wavenumbers were 616, 834, 959, 1096, 1256, 1332, and 3400 cm ^-1^. The TEM image (Fig. 2) also demonstrated that the PVC-NPs exhibited a spherical shape with a particle size < 100 nm. Additionally, the zeta potentials were -9.44 mV, -14.5 mV, and -15.8 mV for PVC-NPs after their exposure to water temperatures of 30 °C, 32 °C, and 34 °C, respectively, confirming that the aggregation of PVC-NPs decreased with increasing temperature (Fig. 3).

### Biochemical analysis

#### Plasma protein, albumin, and globulin levels

As shown in Table 1, the fish groups exposed to 0 and 10 mg/L PVC-NPs among various temperatures showed a significant rise (*p* < 0.05) in plasma protein, albumin, and globulin levels, with the 34 °C groups demonstrating greater values than those at 30 and 32 °C. Besides, compared to those not exposed to PVC-NPs, the levels of all studied parameters increased significantly (*p* < 0.05) across various temperature conditions in the presence of PVC-NPs. The results also showed that plasma proteins, albumin, and globulin concentrations exhibited significant increase (*p* < 0.05) under the interaction effect between the two influences.

**Table 1:** Changes in the biochemical parameters of *O. niloticus* exposed to (0 & 10) mg/L of PVC-NPs at 30, 32, and 34 °C for 4 days.

Parameters	Temperature (°C)	PVC-NPs (mg/L)		Two-Way ANOVA					
				Toxicity		Temperature		Interaction	
		0	10	F	Sig.	F	Sig.	F	Sig.
Total protein (g/dL)	30	3.05 ± 0.09^a^	3.47 ± 0.12^a*^	67.19	< 0.05	35.42	< 0.05	4.07	< 0.05
	32	3.38 ± 0.10^b^	4.11 ± 0.08^b*^						
	34	3.68 ± 0.05^c^	4.77 ± 0.14^c*^						
Albumin (g/dL)	30	0.23 ± 0.02^a^	0.29 ± 0.01^a*^	71.89	< 0.05	47.96	< 0.05	4.96	< 0.05
	32	0.27 ± 0.01^b^	0.34 ± 0.02^b*^						
	34	0.32 ± 0.01^b^	0.47 ± 0.01^c*^						
Globulin (g/dL)	30	2.83 ± 0.09^a^	3.17 ± 0.12^a*^	52.16	< 0.05	33.59	< 0.05	5.06	< 0.05
	32	3.12 ± 0.10^b^	3.77 ± 0.09^b*^						
	34	3.36 ± 0.06^b^	4.30 ± 0.14^c*^						
AST (U/L)	30	54.63 ± 3.85^a^	65.00 ± 1.51^a*^	306.70	< 0.05	301.03	< 0.05	119.40	< 0.05
	32	74.12 ± 2.20^b^	96.69 ± 3.97^b*^						
	34	84.53 ± 1.68^c^	181.13 ± 3.77^c*^						
ALT (U/L)	30	41.94 ± 1.59^a^	76.09 ± 2.22^a*^	498.98	< 0.05	132.64	< 0.05	3.23	NS
	32	58.67 ± 1.15^b^	92.26 ± 1.77^b*^						
	34	70.53 ± 0.78^c^	113.28 ± 3.45^c*^						
ALP (U/L)	30	35.68 ± 1.56^a^	39.68 ± 1.21^a*^	164.72	< 0.05	112.14	< 0.05	25.61	< 0.05
	32	41.75 ± 0.98^b^	59.61 ± 1.82^b*^						
	34	47.39 ± 1.73^c^	72.35 ± 1.46^c*^						
Creatinine (mg/dL)	30	0.30 ± 0.02^a^	0.37 ± 0.01^a*^	111.20	< 0.05	134.38	< 0.05	9.63	< 0.05
	32	0.38 ± 0.02^b^	0.56 ± 0.02^b*^						
	34	0.52 ± 0.02^c^	0.73 ± 0.02^c*^						
Uric acid (mg/dL)	30	3.37 ± 0.06^a^	4.21 ± 0.10^a*^	156.00	< 0.05	125.19	< 0.05	12.33	< 0.05
	32	4.09 ± 0.15^b^	4.63 ± 0.10^b*^						
	34	4.52 ± 0.02^c^	5.95 ± 0.06^c*^						

All values are indicated as mean ± SE (*n* = 5). Data with different letters indicate a significant difference among the different temperatures. Data with star superscripts (*) indicate a significant difference between (0 & 10) mg/L PVC-NPs exposed groups in each temperature.

#### Plasma AST, ALT, and ALP activities

Table 1 displays the changes in the biochemical parameters of the liver functions enzymes (AST, ALT, and ALP) after exposure to 0 and 10 mg/L PVC-NPs at 30, 32, and 34 ^º^C. The results showed that the activities of AST, ALT, and ALP increased significantly (*p* < 0.05) when the fish were exposed to either PVC-NPs or not at the increasing temperature degrees, and the highest elevation arose at groups exposed to 34 ºC when compared to the other water temperatures (30 and 32 ºC). In all temperature conditions, the levels of liver enzymes increased significantly (*p* < 0.05) when exposed to PVC-NPs in contrast to those not exposed to PVC-NPs. Additionally, temperature and PVC-NPs toxicity demonstrated a significant interaction effect (*p* < 0.05) on both AST and ALP activities.

#### Plasma creatinine and uric acid levels

The changes in the kidney’s biochemical parameters (creatinine and uric acid) following exposure to 0 and 10 mg/L PVC-NPs on *O. niloticus* at different water temperatures are shown in Table 1. The concentration of plasma creatinine and uric acid increased significantly (*p* < 0.05) with rising temperature degrees, with the highest increase appearing at 34 °C, particularly in the group exposed to 10 mg/L of PVC-NPs. Moreover, the interaction between temperature and PVC-NPs toxicity showed notable effects (*p* < 0.05) on kidney functions.

### Determination of oxidative stress biomarkers

The alterations in oxidative stress indicators measured in the liver, gills, and brain of *O. niloticus* following exposure to 0 and 10 mg/L of PVC-NPs at varying water temperatures are presented in Tables 2, 3 and 4. Fish exposed to either PVC-NPs or not at increasing temperatures showed a significant increase (*p* < 0.05) in MDA levels. In contrast, GSH and CAT demonstrated the opposite, which decreased significantly (*p* < 0.05) among different temperatures. All oxidative stress biomarkers in the liver, gills, and brain showed that 10 mg/L of PVC-NPs had higher toxic effects, and the maximum toxicity was found at 34 °C water temperature exposure, compared with other temperature degrees. Furthermore, a significant interaction effect (*p* < 0.05) was observed between temperature and toxicity on biomarkers, except for GSH levels in gills.

**Table 2:** Changes in MDA, GSH, and CAT of *O. niloticus* liver exposed to (0 & 10) mg/L of PVC-NPs at 30, 32, and 34° C for 4 days.

Parameters	Temperature (°C)	PVC-NPs (mg/L)		Two-Way ANOVA					
				Toxicity		Temperature		Interaction	
		0	10	F	Sig.	F	Sig.	F	Sig.
MDA (nmol/g. tissue)	30	3.90 ± 0.56^a^	7.03 ± 0.22^a*^	126.03	< 0.05	135.94	< 0.05	18.94	< 0.05
	32	8.24 ± 0.43^b^	13.43 ± 1.19^b*^						
	34	11.60 ± 0.48^c^	23.32 ± 0.99^c*^						
GSH (mmol/g. tissue)	30	87.12 ± 1.49^c^	47.68 ± 1.86^b*^	619.89	< 0.05	170.78	< 0.05	9.72	< 0.05
	32	62.87 ± 1.49^b^	30.45 ± 0.88^a*^						
	34	51.59 ± 1.97^a^	26.21 ± 1.65^a*^						
CAT (U/g. tissue)	30	10.75 ± 0.24^b^	9.94 ± 0.18^c*^	67.49	< 0.05	221.55	< 0.05	8.26	< 0.05
	32	10.20 ± 0.29^b^	8.99 ± 0.13^b*^						
	34	7.14 ± 0.33^a^	4.56 ± 0.12^a*^						

All values are indicated as mean ± SE (*n* = 5). Data with different letters indicate a significant difference among the different temperatures. Data with star superscripts (*) indicate a significant difference between (0 & 10) mg/L PVC-NPs exposed groups in each temperature.

**Table 3:** Changes in MDA, GSH, and CAT of *O. niloticus* gills exposed to (0 & 10) mg/L of PVC-NPs at 30, 32, and 34 °C for 4 days.

Parameters	Temperature (°°C)	PVC-NPs (mg/L)		Two-Way ANOVA					
				Toxicity		Temperature		Interaction	
		0	10	F	Sig.	F	Sig.	F	Sig.
MDA (nmol/g. tissue)	30	4.82 ± 0.29^a^	8.24 ± 0.69^a*^	210.38	< 0.05	44.0	< 0.05	13.08	< 0.05
	32	6.13 ± 0.27^b^	13.22 ± 0.61^b*^						
	34	7.16 ± 0.19^c^	16.06 ± 0.87^c*^						
GSH (mmol/g. tissue)	30	61.20 ± 1.91^c^	44.62 ± 1.23^c*^	203.16	< 0.05	98.99	< 0.05	0.82	NS
	32	47.51 ± 1.71^b^	29.75 ± 1.49^b*^						
	34	41.41 ± 1.94^a^	20.92 ± 0.87^a*^						
CAT (U/g. tissue)	30	9.74 ± 0.25^c^	7.04 ± 0.32^b*^	168.21	< 0.05	103.63	< 0.05	11.11	< 0.05
	32	8.14 ± 0.09^b^	6.76 ± 0.29^b*^						
	34	6.82 ± 0.10^a^	3.14 ± 0.31^a*^						

All values are indicated as mean ± SE (*n* = 5). Data with different letters indicate a significant difference among the different temperatures. Data with star superscripts (*) indicate a significant difference between (0 & 10) mg/L PVC-NPs exposed groups in each temperature.

**Table 4:** Changes in MDA, GSH, and CAT of *O. niloticus* brain exposed to (0 & 10) mg/L of PVC-NPs at 30, 32, and 34 °C for 4 days.

Parameters	Temperature (°°C)	PVC-NPs (mg/L)		Two-Way ANOVA					
				Toxicity		Temperature		Interaction	
		0	10	F	Sig.	F	Sig.	F	Sig.
MDA (nmol/g. tissue)	30	11.04 ± 0.26^a^	19.84 ± 0.72^a*^	555.65	< 0.05	455.77	< 0.05	42.51	< 0.05
	32	18.46 ± 0.44^b^	26.74 ± 0.93^b*^						
	34	24.64 ± 0.58^c^	42.96 ± 0.53^c*^						
GSH (mmol/g. tissue)	30	75.23 ± 2.06^c^	43.29 ± 2.19^c*^	334.16	< 0.05	192.63	< 0.05	4.58	< 0.05
	32	54.85 ± 2.41^b^	23.64 ± 1.85^b*^						
	34	32.45 ± 1.73^a^	11.29 ± 0.54^a*^						
CAT (U/g. tissue)	30	3.71 ± 0.09^c^	3.35 ± 0.11^c*^	36.31	< 0.05	367.27	< 0.05	3.76	< 0.05
	32	3.44 ± 0.08^b^	2.61 ± 0.14^b*^						
	34	1.06 ± 0.06^a^	0.73 ± 0.13^a*^						

All values are indicated as mean ± SE (*n* = 5). Data with different letters indicate a significant difference among the different temperatures. Data with star superscripts (*) indicate a significant difference between (0 & 10) mg/L PVC-NPs exposed groups in each temperature.

## Discussion

Global warming and plastic pollution are the most important environmental problems nowadays. More research is needed to shed more light on the impact of temperature increases on the harmful effects of NPs on aquatic creatures, particularly freshwater species, despite the presence of separate studies on toxicity in both areas^31^. Thus, it has been essential to examine the combined impact of these two distinct problems on *O. niloticus*, the most popular freshwater fish.

The FTIR spectrum exhibits the following vibrational bands and wavenumbers: 616, 834, 959, 1096, 1256, 1332, and the broadband 3400 cm^− 1^, which correspond to the C-H stretching mode, the C-Cl stretching bond, trans-C-H wagging, C-C bond of PVC backbone chain, C-H rocking, the C-H deformation of CHCl, and the O-H hydroxyl bond, which is attributed to the water absorbed by the PVC, respectively. Previous studies have revealed these findings, which confirmed the polymer composition in the current study is PVC^48–50^. TEM image also showed that PVC exhibited a spherical shape with a particle size < 100 nm, proving that PVC is nanosized. Additionally, the zeta potential of particles is used to determine their surface charges. A low value of zeta potential, close to neutral, indicates that particles are more likely to assemble^51^. Pathirana et al.^52^ and Siva et al.^53^ assert that particles aggregate and exhibit diminished stability at low zeta potential levels, whereas elevated zeta potential values inhibit particle aggregation and enhance particle dispersion stability through repulsive forces. Our results showed that all measured PVC-NPs zeta values at different temperatures demonstrated a tendency for particles to aggregate, although the degree of aggregation varied. The zeta value of PVC-NPs at water temperature (30 °C) was nearly neutral and lower, indicating a higher aggregation ability in water compared to PVC-NPs at water temperatures of 32 °C and 34 °C. So, the aggregation of PVC-NPs decreases with increasing water temperatures, which may make them more toxic. According to García-Gómez et al.^54^, the zeta potential of the particle’s suspension varies with the change in characteristics of the medium, which can drastically alter the surface properties, aggregation, and deposition of the particles and affect their environmental behavior and toxicity.

Plasma proteins are essential to fish survival because they regulate numerous processes, including homeostasis regulation and tissue repair^55^. Total protein, albumin, and globulin level estimation are usually used to evaluate fish’s nutritional, metabolic, general health status, and stress status of fish^56^. The current study found that plasma protein, albumin, and globulin levels were increased with increasing water temperature and after exposure to 10 mg/L PVC-NPs. The findings indicate that elevated water temperature and PVC-NPs toxicity may disrupt protein metabolism, resulting in increased plasma protein levels. This alteration in metabolism could be due to either an increase in protein synthesis or a reduction in its degradation rates to counteract these stressors. Kanwal et al.^57^ ascribed the augmentation of protein synthesis to the animal’s ability to react to stressors and the requirement for heightened energy expenditure. Changes in hepatic and renal functioning may indirectly lead to an increase in plasma protein and albumin levels^58,59^. Alterations in hepatic and renal functions were seen in our study; hence, this likelihood could be responsible for the elevation of plasma proteins. Albumin levels also go up because they may act as carriers for PVC-NPs, which makes it easier for them to get to target organs in the fish body, as indicated previously by Banaee et al.^60^. Additionally, the levels of globulin may increase in response to the immune system’s defense mechanisms^59^. Banane et al.^60^ mention that globulin concentrations may also be contingent upon total protein levels. Prior research revealed an increase in plasma protein, albumin, and globulin in fish subjected to elevated temperatures and NPs^61–63^.

Metabolic enzymes such as AST, ALT, and ALP serve as biological indicators for quantifying the degree of stress caused, mainly used to detect liver disorders^64^. In this study, AST, ALT, and ALP increased significantly in response to temperature and PVC-NPs exposure. This suggests that hepatic damage was exerted, and enzymes were released into the blood by elevated temperature and PVC-NPs toxicity after the destruction of cell membranes or intracellular organelles^11,65^. A similar finding has been reported by Iheanacho and Odo^27^, who stated that the rise of AST and ALT levels in *Clarias gariepinus-*treated groups with PVC was due to liver injury. Various studies have reported elevated levels of liver enzymes in different fish species exposed to MPs, such as *Pseudobagrus fulvidraco*,* O. niloticus*, and *Danio rerio*^55,59,66^. A rise in AST and ALT activities has also been seen in *Dicentrarchus labrax* and *Neolissochilus hexagonolepis* when exposed to high temperatures^67,68^. They suggested that the fish may have liver dysfunction due to increasing temperatures. Moreover, the present results revealed the highest elevation of liver enzymes at 34°C after exposure to PVC-NPs, suggesting a synergistic effect between the increasing temperature and PVC-NPs exposure leading to more toxic effects. This finding agrees with what Gholamhosseini et al.^11^ found. They demonstrated that *Astacus leptodactylus* exhibited the highest AST, ALT, and ALP activities when exposed to PE-MPs at higher temperatures.

The assessment of kidney function can be effectively determined by measuring the levels of creatinine and uric acid in the plasma, which serve as reliable indicators of glomerular filtration rate and renal failure^69^. Toxicants elevate the generation of reactive oxygen species (ROS), impairing biological processes and systems, hence hindering renal function and affecting the excretion of uric acid and creatinine^58,70,71^. This study showed that plasma levels of creatinine and uric acid went up. This rise may be linked to kidney damage caused by PVC-NPs’ toxicity by increasing ROS production. Following exposure to MPs, Hamed et al.^59^ and Banaee et al.^72^ previously observed an elevation in the levels of creatinine and uric acid in *O. niloticus* and *Oncorhynchus mykiss*, which was associated with renal impairment. Furthermore, the current data showed an increase in creatinine and uric acid as temperatures rose, potentially due to renal injury too. As indicated by Yang et al.^73^ and Dawood et al.^74^, higher temperatures resulted in elevated plasma creatinine levels in *Scophthalmus maximus* and *Clarias gariepinus*, which are associated with kidney damage. Also, such an elevation in creatinine may be due to the increase in creatine helping to supply more energy to muscles after increasing water temperature and/or exposure to PVC-NPs^75^.

The influence of increasing temperature in the presence of PVC-NPs amplifies the toxic effect on biochemical parameters, including total protein, albumin, globulin, AST, ALT, ALP, creatinine, and uric acid, more than the effect of increasing the temperature alone. This confirms the synergistic effect between increasing temperatures and PVC-NPs.

Oxidative stress biomarkers are used to assess how different environmental stressors affect aquatic organisms. According to Hu and Palić^76^ and Pei et al.^77^, after penetrating the bloodstream, NPs or MPs may enter the cells, causing NADPH oxidase to produce ROS, and the higher ROS levels boost the antioxidant system. Furthermore, elevated temperatures and hypoxic conditions can trigger oxidative stress, leading to an increased generation of ROS in cells^78^. Fish have a detoxifying ability that aids in the elimination of ROS toxicity^79^. Nevertheless, if not promptly eradicated, the remaining balance will lead to oxidative damage^69^. The liver’s function is responsible for filtering harmful substances from the body, and gills, being constantly exposed to water, are employed to test the fish’s ROS-raking abilities^80^. Additionally, the brain’s strong oxidative metabolism and quantity of polyunsaturated fatty acids in cell membranes make it especially vulnerable to oxidative damage, which manifests as ROS generation^81^. So, liver, gills, and brain were selected to demonstrate how elevated temperatures and PVC-NPs can induce oxidative stress in these tissues.

MDA, GSH, and CAT are oxidative stress biomarkers; studying them can provide a useful model for investigating the possible impact of oxidative stress on cell activities^82,83^. The amount of MDA, a byproduct of lipid oxidation, can be used as an indirect measure of oxidative damage^84^. According to Dawood et al.^74^, prolonged stress and extreme conditions lead to a significant buildup of ROS that are implicated in the oxidation of lipids, as indicated by the elevated levels of MDA. The current study revealed elevated MDA levels in the liver, gills, and brain of *O. niloticus* when exposed to varying temperatures. These levels increased as the temperature increased, indicating that an increase in water temperature can trigger more ROS and lead to higher lipid peroxidation. Ali et al.^85^ found similar results, observing that the peroxidation of the plasma membrane in response to temperature stress was demonstrated by the considerable increase in MDA in the *Dicentrarchus labraxh* groups when exposed to 33 °C and a dramatic increase at 36 °C. The results also showed an elevation in MDA after exposure to PVC-NPs, suggesting that NPs can cause oxidative stress and problems with the body’s antioxidant defenses. Another study by Banaei et al.^86^ found that MDA levels rose in the liver cells of the fish *C. carpio* when it was exposed to different amounts of PE-MPs. Additionally, such elevation in the liver and brain was recorded by Xia et al.^87^ in *Paramisgurnus dabryanus* exposed to PE-MPs. A gradual increase was observed in MDA activity of liver and gill tissues of *Hypophthalmichthys molitrix* after PS-NPs exposure, too^88^.

GSH is an always-present, non-enzymatic antioxidant that plays a role in detoxification because of its ability to remove ROS which protects cells from oxidative stress directly^89^. The current study reveals that GSH levels in the liver, gill, and brain of *O. niloticus* were significantly diminished after exposure to PVC-NPs and increased temperature, indicating that these stressors induce substantial oxidative stress, resulting in a more rapid depletion of GSH to counteract the surplus ROS. Reduced GSH levels are linked to increased ROS formation in the presence of MPs in tissues^22^. Huang et al.^90^, Atamanalp et al.^91^ and Lee et al.^55^ also showed that exposure to PS-MPs and PE-MPs significantly decreased GSH levels in different tissues of *Poecilia reticulata*, *O. mykiss* and *Pelteobagrus fulvidraco* by causing an imbalance in the antioxidant defense system. Moreover, thermal stress, in conjunction with chemical impacts, causes changes in GSH concentrations in aquatic organisms^92,93^. Freitas et al.^94^, for example, showed that all groups of *R. schneideri* tadpoles treated with clomazone at higher temperatures had lower GSH levels than those treated at lower temperatures. Our data also indicated that PVC-NPs reduced GSH levels as temperatures increased, suggesting that the amalgamation of these stressors led to heightened ROS and a disruption in antioxidant mechanisms.

CAT activity gets rid of free hydrogen peroxide radicals (H_2_O_2_) as a defense against outside sources of superoxide^83^. This makes it a key indicator for figuring out how toxic something is to fish. According to our results, the higher H_2_O_2_ under PVC-NPs and the temperature effect may have imbalanced the antioxidant system, inhibiting CAT activity in the studied tissues of the exposed groups. Unfortunately, this reduction may result in insufficient clearance of excessive H_2_O_2_, which could accumulate in cells and cause more oxidative damage^65^. A reduction in catalase activity was previously reported in *Cyprinodon variegatus* exposed to 50 mg/L PE-MPs^95^. Furthermore, Espinosa et al.^96^ observed reduced liver CAT activity of *Dicentrarchus labrax* after exposure to different MPs. Iheanacho and Odo^28^ also observed the inhibition of CAT activity in the brain of *C. gariepinus* after PVC-MPs exposure. Martyniuk et al.^97^ demonstrated a reduction in CAT activity in the freshwater mussel *Unio tumidus* upon exposure to PS-MPs at an elevated temperature too.

All the current oxidative stress biomarker responses by PVC-NPs got worse as the water temperature went up. The most harmful effects were seen when fish were exposed to water at 34 °C, which means a combination of temperature and PVC-NPs can cause an imbalance in cellular redox homeostasis, increasing ROS and leading to more oxidative damage. This was in line with what Park et al.^98^ found, which is that the upregulation of antioxidant defense by Pb was stronger at a temperature higher (34 °C) than the rearing temperature (26 °C).

Finally, all current data demonstrated that the fish would experience adverse effects from temperature increases of 2 and 4 degrees, as predicted by the future global warming scenario. Additionally, in the presence of PVC-NPs, the toxicity increases with increasing temperature, surpassing the effect of temperature alone. This could be attributed to its influence on the surface properties of PVC-NPs, as evidenced by our zeta potential results, which indicate that increasing temperature reduces aggregation and increases the solubility of PVC-NPs, increasing their toxic effects. According to Wang et al.^99^, the toxicity of particles is generally inversely proportional to their size; aquatic organisms find the less aggregated NPs more bioavailable, making them more toxic. Also, the lack of oxygen that comes with rising temperatures can make PVC-NPs more bioavailable and help them build up in living tissues, as Kibria et al.^100^ found for other pollutants. The present results approved that combining stressors like PVC-NPs and temperature puts *O. niloticus* under more stress due to their synergistic effect. Several studies highlighted previously that thermal and pollutant stressors can induce a synergistic broad range of adverse effects on different species^1,31,75,98,101^.

## Conclusions

Few studies have examined the impacts of NPs compared to MPs on aquatic organisms, particularly those at higher trophic levels, like fish, and how the negative effects of plastics on freshwater species change as a function of increasing water temperatures. This study is the first to investigate the harmful effects of virgin PVC-NPs and it shows how higher temperatures change the toxicological properties of PVC-NPs on different health biomarkers of *O. niloticus*. The results showed that increasing water temperature caused significant biochemical and oxidative stress changes in fish. Moreover, the current study found that the presence of PVC-NPs, particularly at 34 °C, intensified the alterations and harmful impacts on fish. As a result, we should not disregard the expected global warming scenario, particularly considering other global issues such as plastic pollution. Aquatic organisms living in a polluted ecosystem may be more or less healthy depending on the surrounding conditions. So, more research is needed to assess the effects of other virgin NPs on various fish species, particularly as temperatures rise due to the anticipated global warming scenario.

## Data Availability

The datasets used and/or analyzed during the current study are available from the corresponding author on reasonable request.
